# Inequalities in health and health-related indicators: a spatial geographic analysis of Pakistan

**DOI:** 10.1186/s12889-020-09870-4

**Published:** 2020-11-26

**Authors:** Sami Ullah Khan, Ijaz Hussain

**Affiliations:** Department of Economics, Gomal University, Dera Ismail Khan, Pakistan

**Keywords:** Community health, Human development index, Disparities, Socioeconomic indicators, Spatial analysis, Pakistan

## Abstract

**Background:**

In developing countries, Pakistan is one of the countries where access to health and health-related indicators is a major concern. Their improvement would reduce inequalities among various Communities/Districts or groups of Communities. A Community health index (CHI) in this regard is estimated to explore inequality ratio, inequality slope, and spatial analysis of inequalities among all Communities at regional and geographical levels.

**Methods:**

Data from Pakistan Social and Living Standard Measurement (PSLM) survey, Round-VI, 2014–15 were used to construct CHI. The index was constructed in two steps. In the first step, the study indicators were standardized while in the second step, the standardized indicators were aggregated into a single metric by applying non-linear Geometric Mean formula.

**Results:**

The inequality ratio of 16.59 estimated for Pakistan was found to be higher than the ratio of Atlanta city, GA (5.92), whereas, a lower slope coefficient was estimated for Pakistan than Atlanta city, GA (0.38 < 0.54). This ratio of disparity was also found to be lower for urban regions as compared to rural (7.78 < 17.54). While the slope coefficient was slightly higher for urban regions (0.45 > 0.43). The results of the spatial analysis revealed different patterns of inequalities. A cluster of healthy districts was found in Punjab province, whereas districts from Baluchistan had made a bunch of deprived/unhealthy districts in terms of CHI scores. Besides, separate maps for all provinces showed that capital districts of all provinces were relatively well-off/developed.

**Conclusion:**

The instant results concluded that inequalities in access to health and health-related indicators exist across countries as well as across geographical regions. To reduce or eradicate these inequalities, government and public health workers are recommended to set priorities based on access to composite index.

**Supplementary Information:**

The online version contains supplementary material available at 10.1186/s12889-020-09870-4.

## Background

Improvement towards equitable access to health and health-related determinants is a global concern and fundamental to the advancement of Sustainable Development Goals (SDGs) [1]. Access to health-related social indicators is one of the major subjects playing a pivotal role in the determination of economic growth [2, 3]. On the other hand, rapid urbanization and a growing population have threatened people’s accessibility to health and the environment. About 400 million people have no access to basic health facilities and nearly 7 million people die each year due to air pollution mostly in low and middle-income countries [4, 5]. Likewise, more than 15 million people living with HIV have no access to treatment, and it is evident that every 2 s, people aged 30 to 70 years die prematurely from non-communicable diseases, chronic respiratory disease, diabetes, and cancer [1, 6]. Challenges like increasing population and growing urbanization not only influenced the basic health care facilities but have also narrowed the accessibility to other health/wellbeing related factors. Sociodemographic factors of income and education, housing-related indicators, geographical and environmental attributes are considered as a range of significant social determinants of health which are multiple and interactive [7–12].

Nations throughout the globe are lacking access to health and health-related indicators, but more in low- and middle-income countries. According to UNDP’s report, about 103 million of the world population has no literacy skills [1]. Nearly 57 million primary-aged children are out of school, and, in low- and middle-income countries each fourth girl is not enrolled in school [1, 13]. Approximately 844 million people have a lack of access to safe drinking water and about 2.3 billion world population has no access to sanitation facilities [1]. Because of continuous population growth, the demand for cheap energy is increasing and the society relying on fossil fuels is creating severe changes to the environment. It is worth mentioning here that one in seven people has a lack of access to electricity facility and the majority of these people are living in rural areas of developing countries [14]. Moreover, about 3 billion people use solid fuels such as wood, charcoal, crop waste, and dung for cooking practices, most of them are living in low- and middle-income countries [4]. Similarly, increasing population and rising migrations have boosted urbanization, especially in developing countries which resulted in slums as a significant issue of urban life. In 2018, 4.2 billion world population lived in cities and is expected to increase by 90% in developing countries [1].

Pakistan being a low- and middle-income country faces a lack of access to health and health-related socioeconomic indicators. About 39% of the population suffers from multi-dimensional poverty [15]. Considering the Maternal Mortality Rate Index (MMRI), in 2015, Pakistan ranked at 149 out of 179 countries [16]. Approximately 20% of children aged 6 to 16 years do not have access to school [17]. Access to the quality of drinking water has also deteriorated. Pakistan ranks at the top 9th position with the lowest access to safe drinking water and only 20% of the whole population of Pakistan has access to clean drinking water [18–20]. Most people in Pakistan use solid fuels such as wood, charcoal, crop waste, and dung for cooking practices and only 22% of the population has access to natural gas as a clean cooking fuel [21].

It could be viewed as access to health and health-related socioeconomic, housing, clean drinking water, and environmental factors, the government of Pakistan has made extraordinary progress in accessibility and sustainability towards these indicators since 2000 [22]. However, there is substantial geographic heterogeneity in access to health and health-related factors [22]. There is a need to measure the extent of accessibility/sustainability and geographical inequalities/disparities towards these indicators, as health and health-related social indicators are connected with UNDP’s Sustainable Development Goals (SDGs). SDG’s are integrated and improvement in one or more dimensions will enhance the overall economic development of a country [23].

There are several approaches used to measure geographical disparities. One of these popular approaches is the recently developed Urban Health Index (UHI) [24]. Though this framework/tool is not predicated on a new method, rather it is based on the well-known approach of Human Development Index (HDI) [24]. In the present study, we applied the UHI tool based on certain silent features. First, the main characteristic of the index is that it aggregates various dimensions in a single metric to know the extent of development/accessibility. Second, The UHI tool is useful as it allows users to choose the scale/unit of analysis (from a small area to international level) as well as freedom of indicators (mainly depend upon data availability) and mode of presentation. More importantly, the single value of the index is advantageous in measuring the spatial disparities in the health/well-being of cities at different geographical levels [24, 25]. Recently, various studies have applied the UHI tool to obtain their objectives [25–29]. Keeping in view the above facts, in this study we have constructed a complex index named Community Health Index (CHI) to measure the disparity ratios, disparity slopes, and patterns of spatial geographical inequalities among districts of Pakistan.

As the aim of the composite index (Community Health Index) is to examine health/wellbeing disparities among districts at the geographical level, this paper can contribute to the existing literature from certain perspectives. First, the composite index is constructed very rarely and has not been constructed before in Pakistan. Second, in Pakistan, there has been no study found on district health/wellbeing disparities at the regional and geographical levels. Finally, spatial analysis of health inequalities has not been conducted in Pakistan and we have analyzed the data spatially through ArcGIS and GeoDa applications [30, 31].

## Methodology

Before explaining the step-wise methodology of CHI, the selection of appropriate domains (and their sub-domains) was the main problem in index construction. Therefore, the dimensions were selected based on certain important grounds. First, based on previous literature these indicators have significantly influenced the health status [10, 27, 32–40]. Second, these indicators were expected to be positively correlated with one another. Keeping in view these facts, eight indicators from five dimensions were chosen. The detailed description is given in Table 1 below.

**Table 1:** Description of selected Dimensions and their sub-dimensions of the study

S. No	Dimension	Sub-dimension
1	Economic domain	i. Mean income of households per year (in rupees)
2	Education domain	i. Households head with high school degree or higher (in percentage) ii. Households spouse with middle school degree or higher (in percentage)
3	Housing domain	i. Households using Gas as a fuel for cooking purposes (in percentage)
4	Water domain	i. Households with piped water as a source of drinking water (in percentage)
5	Health domain	i. Households where lady health worker (LHW) visited in last 30 days ii. Children under 5 who have completed the full recommended vaccination course (in percentage) iii. Households receiving post-natal care after delivery (in percentage)

Source: “Pakistan Social and Living Standard Measurement survey”, 2014–15

“()” shows the measurement scales

### Construction of Community Health Index (CHI)

To construct the Community Health Index (CHI), the WHO’s methodology was applied, recently used to measure the Urban Health [24]. CHI was constructed in two steps.

#### Step1. Standardization of indicators/sub-dimensions

The selected indicators were standardized by taking the difference of the indicator’s actual value from its lowest value divided by the difference between the highest values to the lowest value. Lowest and highest values are also known as the minimum and maximum goalposts respectively. The mathematical derivation is given in Eq. (2.1) as under;

2.1 $$ {I}^s=\frac{I-{\mathit{\min}}^{\ast }(I)}{\mathit{\max}(I)-{\mathit{\min}}^{\ast }(I)} $$

Where; *I* = actual value of an indicator.

Max (*I*) = maximum value of indictor *I.*

Min* (*I*) = minimum value of indicator *I* minus a small value or a chosen value.

*I*^*s*^ = calculated standardized value of indicator *I,* satisfying the 0 < *I*^*s*^ ≤ 1 condition.

#### Step2. Aggregation of standardized indicators into a single metric

In the second step, the standardized indicators were amalgamated through non-linear aggregation (Geometric Mean) formulae. The Geometric Mean methodology is explained mathematically in Eq. (2.2) as under;

2.2 $$ CHI={\left[\prod \limits_{i=1}^j{I_i}^s\right]}^{\frac{1}{j}} $$

Where “*j*” is the total number of indicators and “1/j” is the power of the product of all standardized indicators. The index score was calculated by multiplying the values of standardized indicators together for all communities (districts) and raising the product to the *j*^*t*h^ root. This simple Geometric Mean formula assigned equal weights to all sub-dimensions or simply it is known as Unweighted Geometric Mean. However, as in the present study, all dimensions (and not their sub-dimensions) were assumed to be weighted equally, the above simple Geometric Mean formula had been converted to the Weighted Geometric Mean formula. The mathematical expression of Weighted Geometric Mean is written in Eq. (2.3) below;

2.3 $$ CHI={\left[\prod \limits_{i=1}^j{sI}_i^{w_i}\right]}^{1/{\sum}_{i=1}^j{w}_i} $$

Where “*w*_*i*_” are the weights of selected indicators. These weights were assigned/scaled so that their sum equals the total number of indicators. By doing so, the power of Weighted Geometric Mean formula ($1/{\sum}_{i=1}^j{w}_i$) will simply become ($\frac{1}{j}$).

### The ratio of disparity

The disparity ratio (inequality gap) was computed by taking the ratio of the mean of the upper 10% of the distribution (first decile) to the mean of the lower 10% of the distribution (last decile) of CHI scores. Mathematically we can write the formula as under;

2.4 $$ Dispa\mathrm{r} ity\  Ratio=\frac{Mean\ of\ the\ upper\ 10\% of\ distribution}{Mean\ of\ the\ lower\ 10\% of\ distribution} $$

### The slope of disparity/inequality

The disparity/inequality slope of the middle 80% of CHI scores was estimated by applying simple linear regression through the Ordinary Least Square (OLS) technique. These districts/communities were ranked in ascending order in terms of CHI scores. The CHI scores were regressed on the rank variable. Mathematically;

2.5 $$ {\mathrm{C} HI}_i=f\ \left({Rank}_i\right) $$

Where “CHI_i_” is the index score of “i^th^” district and “Rank_i_” is the rank of that particular district. “*f*” shows a functional relationship between the dependent and independent variables. Equation (2.5) is econometrically be written as under;

2.6 $$ {CHI}_i=\alpha +\beta {Rank}_i+{\varepsilon}_i $$

In Eq. (2.6), “*β*” is the disparity slope that shows the average CHI heterogeneity between each adjacent district (Community) and is influenced by the ranking of districts/communities. As the number of communities was varied in different provinces, the rank variable was rescaled through dividing each area’s rank by the total number of units in the midsection and regressed the CHI scores again on the rescaled rank variable.

2.7 $$ {CHI}_i=\alpha +\beta Resca\mathrm{l} ed{Rank}_i+{\varepsilon}_i $$

The slope estimated through Eq. (2.7) can be used to compare two regions with a varied number of areas.

### Visualization of CHI scores on maps

As one of the objectives is to assess the patterns of spatial geographical disparities among districts of Pakistan, the CHI scores were displayed on choropleth maps. Spatial representation is useful because one can directly differentiate the extent of the health/well-being quality of various districts for different regions. Moreover, it provides essential information about different patterns of health (well-being) to public health workers and can make some useful planning in this regard. A step by step brief procedure of how to show CHI scores on maps is given in Additional file 1.

### Data

Data were extracted from Pakistan Social and Living Standard Measurement Survey (PSLM): Round-VI, 2014–15 conducted by the Pakistan Bureau of Statistics (PBS), Islamabad [41]. This survey was one of the core instruments to implement the development projects and to track the Millennium Development Goals (MDGs). It provided a detailed information about social factors at the district level. The survey consisted of all urban and rural areas of the four provinces of Pakistan and Islamabad (international capital territory) as well. Pakistan Bureau of Statistics (PBS) had adopted the stratified two-stage sample design methodology and the population of all provinces was considered a universal sample. In the framework of the PSLM survey, all cities/towns were subdivided into enumeration blocks. The sample size for the present study was fixed at 78465 households covering 5428 enumeration blocks. Among these, data were collected from 5326 blocks and the rest were dropped due to the law and order situation [41]. These enumeration blocks were expected to produce consistent results at the district level.

### Statistical tools

For analysis purposes, Excel spreadsheets (Microsoft Excel®, Microsoft Corporation), Statistical Package for Social Sciences (SPSS) version 20, Geographic Information System (ArcGIS), and GeoDa applications were used throughout the study [30, 31].

## Results

As the study was based on the assumption that the selected indicators should be positively correlated with one another, Pearson’s Correlation Metrix is shown in Table 2. The table shows that all the indicators were positively correlated with one another except “Piped Water” and “Health domain”. However, slightly negative correlations are usually acceptable [24]. All these associations were significant except “Piped Water” to “LHW visit” and “Post-natal care”.

**Table 2:** Correlation between selected indicators (Pearson’s Correlation Metrix)

	1	2	3	4	5	6	7	8
**1. Mean Income**	1							
**2. Head Education**	.48^a^	1						
**3. Spouse Education**	.55^a^	.62^a^	1					
**4. Gas Facility**	.43^a^	.65^a^	.73^a^	1				
**5. Piped Water**	.26^a^	.18^c^	.19^b^	.23^b^	1			
**6. LHW visit**	.16^c^	.13^c^	.41^a^	.16^c^	−.24	1		
**7. Vaccination**	.40^a^	.21^b^	.63^a^	.36^a^	−.18^c^	.64^a^	1	
**8. Post-natal Care**	.23^b^	.33^a^	.35^a^	.36^a^	−.001	.23^c^	.33^a^	1

Data source: Author’s own computations based on “Pakistan Social and Living Standard Measurement survey”, 2014–15

“^a^”, “^b^” and “^c^” denotes level of significance at 1% and 5% and 10% respectively

### Disparity ratio and disparity slope of Pakistan

**Fig. 1:**
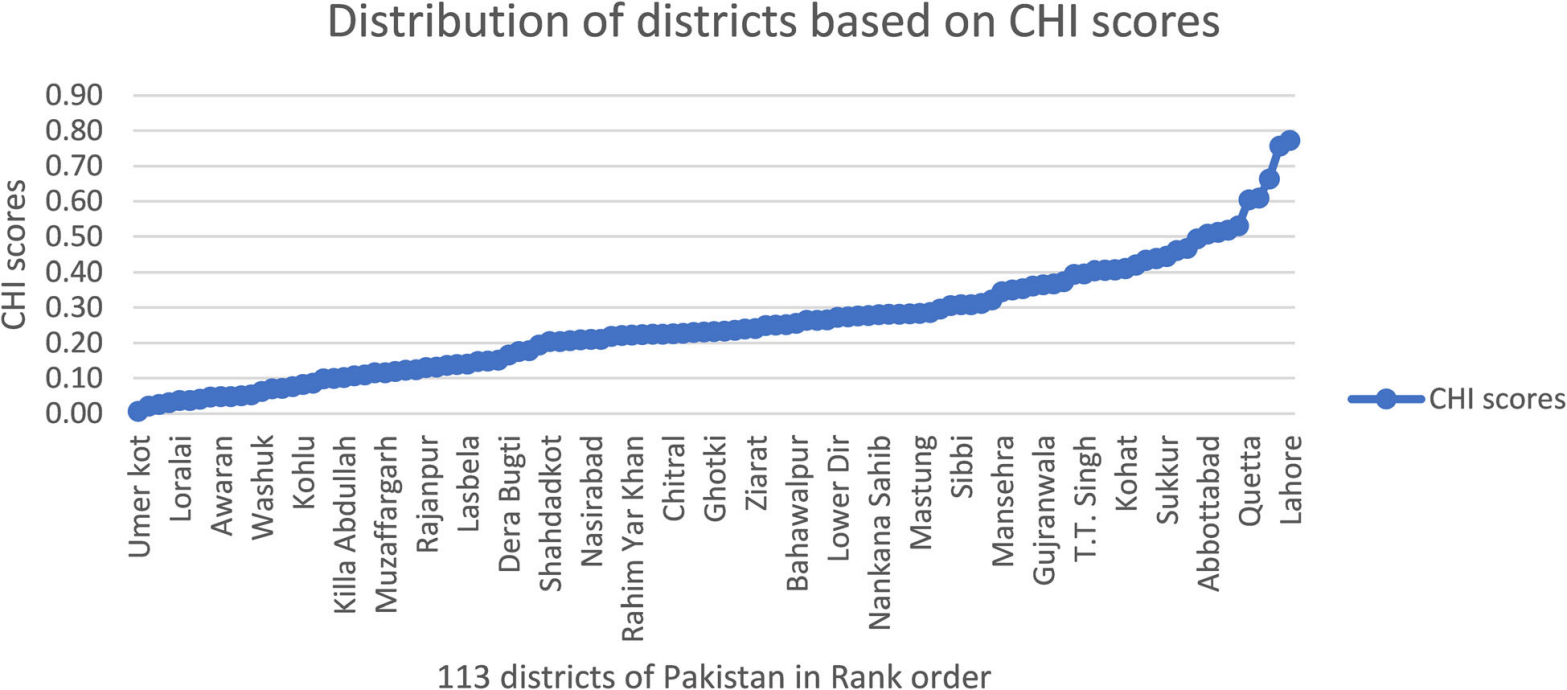
Distribution of Community Health Index (CHI) for districts of Pakistan in year 2014–15. Source: Author’s own computations based on “Pakistan Social and Living Standard Measurement (PSLM) survey”, 2014–15. CHI scores are Community Health Index (Standardized) values

The distribution of the Community Health Index (CHI) for 113 districts of Pakistan is shown in Fig. 1. The disparity ratio (ratio of extremes) for this distribution was calculated as 16.59. It depicted that in terms of CHI scores the upper decile districts were 16.59 times healthier/more developed than lower decile districts of distribution. District “Umerkot” was considered as a less-developed (highly deprived) district of Pakistan, whereas “Lahore” was the healthiest/developed district in terms of CHI scores. The detailed list of all districts lies in upper and lower deciles along with their concerned CHI scores is given in Additional file 2, Table A. The disparity slope estimated for the mid-section districts (middle 80% of the districts) was found to be 0.38 (Additional file 2, Table B). On average, the adjacently ranked districts changed by 0.38 CHI units with a unit change in districts ranking.

### Spatial representation of the district’s CHI scores of Pakistan

**Fig. 2:**
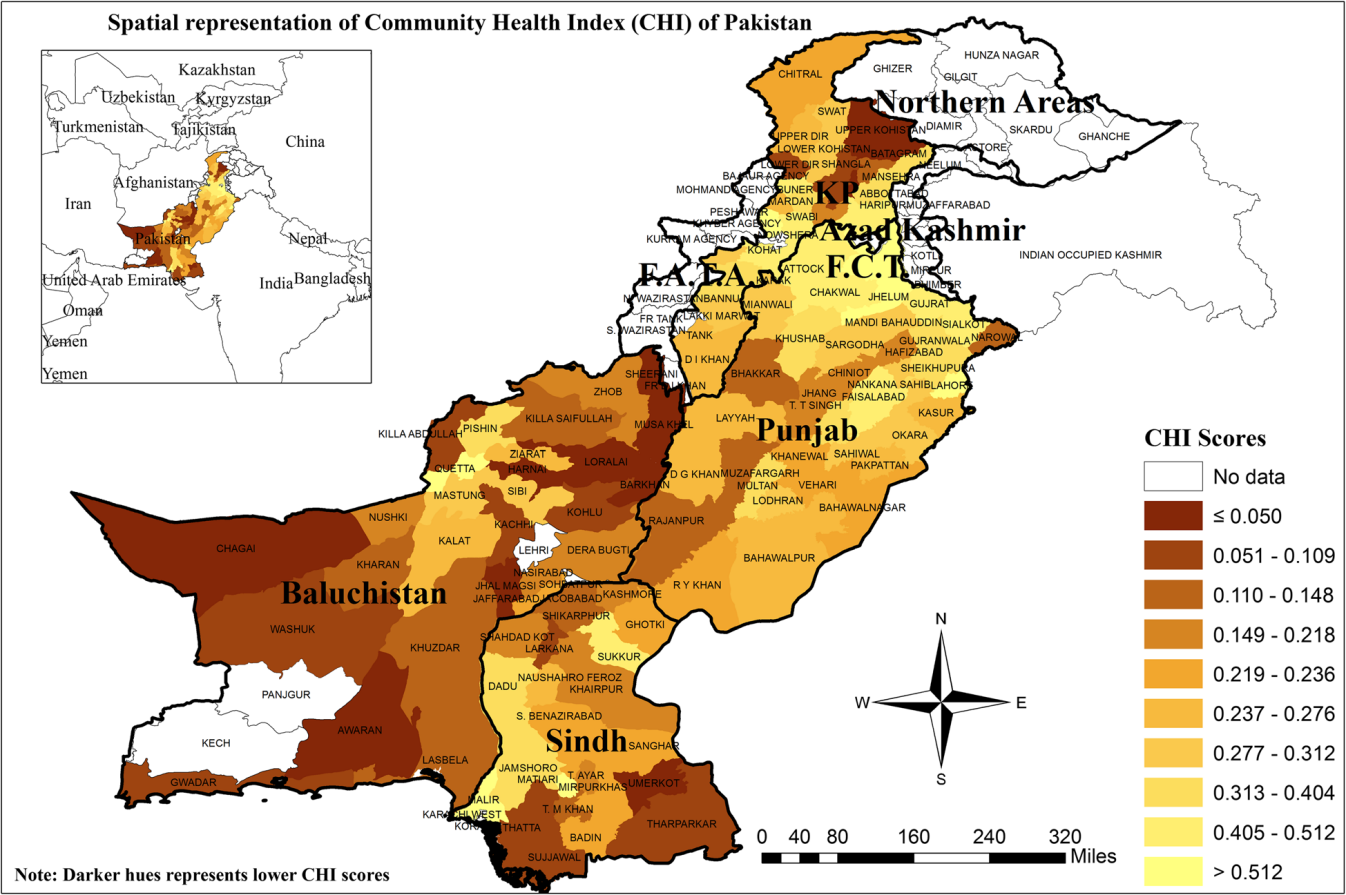
Spatial representation of Community Health Index (CHI) scores of Pakistan in year 2014–15. Source: Author’s own computations based on “Pakistan Social and Living Standard Measurement (PSLM)survey”, 2014–15. CHI scores are used to generate the map through ArcGIS, version 10.5 software

Figure 2 provides a visual representation of CHI scores on a map to assess the spatial patterns of inequalities in Pakistan. The map clearly showed that the pattern is becoming brighter as we move from the “West-Southern” districts to the “East-Northern” districts. The pattern also displayed a cluster of multidimensionally unhealthy districts from Baluchistan province. However, some less developed districts were from both Khyber Pakhtunkhwa (KP) and Sindh provinces. On the other hand, a cluster of relatively healthier districts in terms of CHI scores was found in Punjab province. An interesting outcome here is that the capital cities (Lahore, Karachi, Peshawar, and Quetta) of all provinces were ranked in top districts of Pakistan.

### Urban/rural comparison based on disparity ratio and disparity slope

**Fig. 3:**
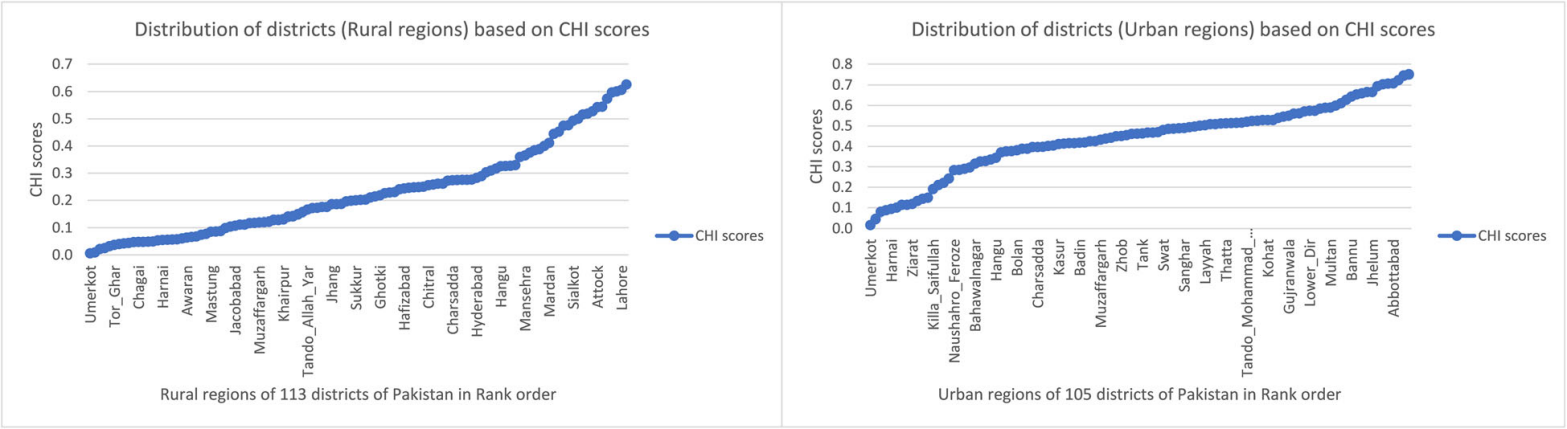
Distributions of Urban/Rural region’s CHI scores of all districts. Source: Author’s own computations based on “Pakistan Social and Living Standard Measurement (PSLM) survey”, 2014–15 CHI scores are Community Health Index (Standardized) values≤

Figure 3 illustrates the CHI score’s distributions of urban and rural regions. The disparity ratios for urban and rural regions were recorded as 7.78 and 17.54 respectively. Considering the urban regions, “Peshawar” was the healthiest while “Umerkot” was the most deprived community/district in terms of CHI score. Likewise, in the rural region, “Rawalpindi” and “Umerkot” were respectively the relatively developed and deprived districts of Pakistan. The detailed list of upper and lower decile (both for urban and rural regions) districts with CHI values is given in Additional file 2, Table C. Further, the disparity slope for urban regions was estimated as 0.45, while for rural regions it was 0.43 (Additional file 2, Table D). The urban and rural regions on average changed by 0.45 and 0.43 CHI units correspondingly with a change in contiguously ranked communities.

### Spatial representation of CHI scores of urban/rural regions

**Fig. 4:**
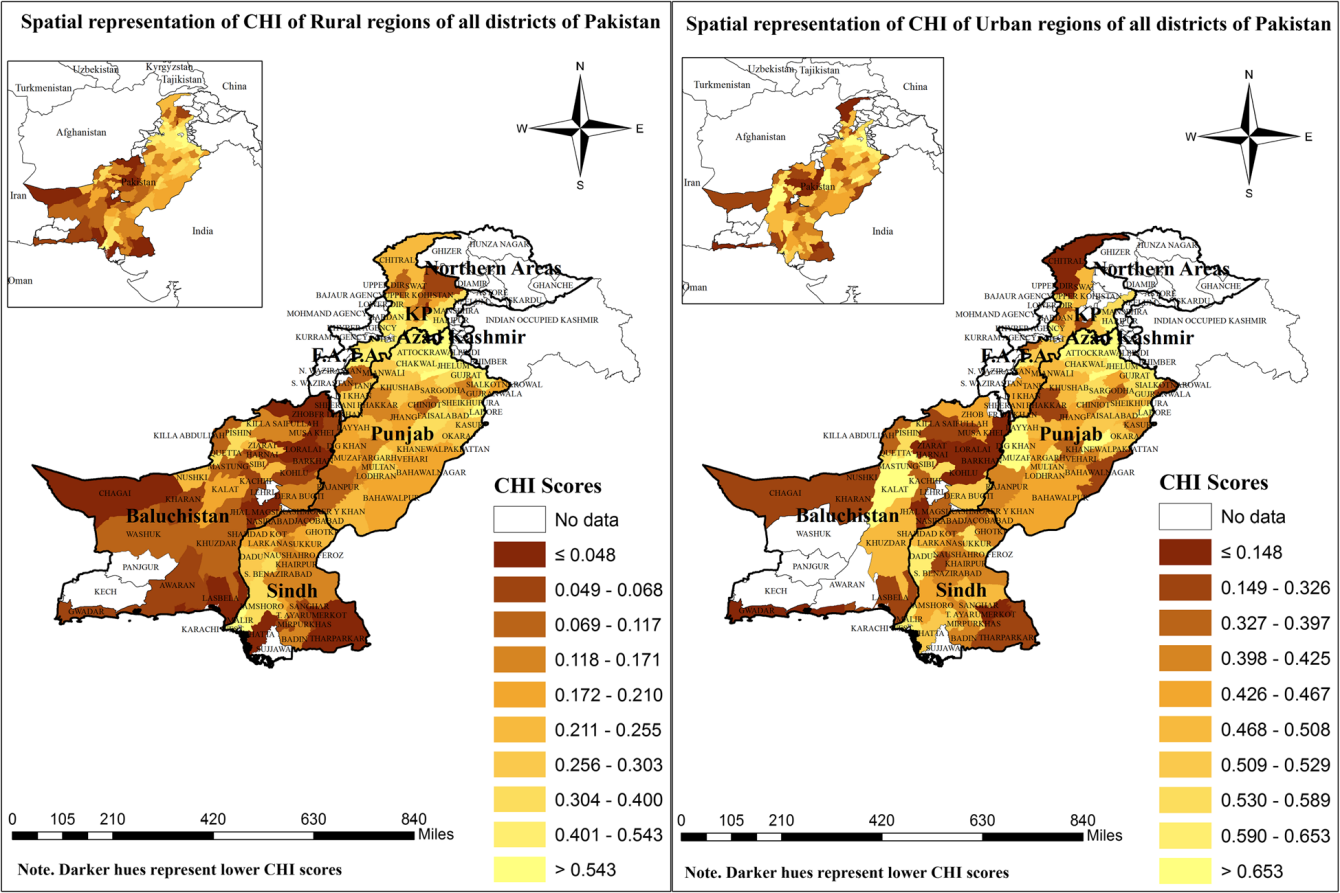
Spatial representation of CHI of urban and rural regions of all districts of Pakistan. Source: Author’s own computations based on “Pakistan Social and Living Standard Measurement (PSLM) survey”, 2014–15. CHI scores are used to construct the map through ArcGIS, version 10.5 software

Figure 4 shows considerable variations between urban/rural spatial patterns of inequalities. Overall, the patterns showed that the urban regions were well-off in terms of CHI scores than rural regions as the hues of urban regions were slightly brighter. The right panel of Fig. 4 (urban) displayed that majority of the districts from Baluchistan province were relatively worse-off/unhealthy. The same is true for the rural region (left panel of Fig. 4).

## Discussion

There has been a significant improvement in access to health and health-related social indicators but inequalities exist across nations as well as across different geographical regions within the country. For instance, in our study, the disparity ratio (16.59) is higher than the ratio of 5.92 found for census tracks of city of Atlanta, state of Georgia, USA [25]. Comparative to city of Atlanta GA, the multidimensionally developed districts of Pakistan are far away from their undeveloped counterparts. In other words, upper decile districts are enjoying nearly all health-related social facilities than lower decile district. The results also confirmed a considerable difference between the disparity ratios of urban and rural regions. The distance/gap between developed and deprived districts in terms of CHI scores is less for urban regions as compared to rural (7.78 < 17.54). Regarding access to health and their related determinants, there is a higher unequal distribution in rural than in urban regions of the districts. These inequality ratios are also varying among various provinces. The lower disparity ratio (4.78 < 12.51, 16.61, and 15.35 respectively for KP, Sindh, and Baluchistan) of Punjab province clearly showed that the upper and lower decile districts are less unequally distributed than other provinces (Additional file 3, Figure A).

There is also a significant difference in disparity slopes across nations as well as across various geographical groupings within a country. Census tracks of city of Atlanta, GA are more heterogeneous and sensitive (with a slope of 0.54 [25]) than districts of Pakistan (having a slope coefficient of 0.38). Access to complex index (CHI) is enhanced by 0.38 CHI units with an increase in the ranking of contiguous districts. Both urban and rural regions are sensitive. However, this heterogeneity is found to be slightly higher in urban regions than in rural (0.45 > 0.43). Further, like urban/rural differences concerning inequality slopes, it is also obvious from results that Punjab province has the lowest disparity slope (0.263) as compared to other provinces (Additional file 2, Table E).

Spatial analyses are useful in understanding the patterns of inequalities among various geographical regions. Figure 2 displayed the spatial differences among all study districts in terms of CHI scores. It is obvious from the map that Northern and Eastern districts are relatively healthier/developed. These districts are mostly of Punjab and Khyber Pakhtunkhwa (KP) provinces. A cluster of unhealthy districts is found in the Western and Southern regions of Pakistan, which means that these districts have no proper access to health and health-related social factors. Moreover, from the spatial patterns, it is clear that people in Punjab are enjoying the health and their related social indicators while the majority of Baluchistan’s people are suffering from lower accessibility to these facilities. Capital districts of all provinces (Peshawar from KP, Quetta from Baluchistan, Karachi from Sindh, and Lahore from Punjab) are highly developed.

To assess whether there exist variations in the spatial pattern of urban and rural regions of all districts, separated maps for both rural and urban regions have been constructed (Fig. 4). From both patterns, it is apparent that urban regions have higher access to multi-dimensional Index (CHI). Further, the patterns of Baluchistan province displayed that rural regions of Baluchistan are worse-off than urban regions. It is also obvious from CHI scores of each decile shown in the legends. All deciles of urban regions have higher CHI scores than urban regions.

A separate spatial analysis of each province provided information about where the clusters of healthier or unhealthier districts in terms of CHI scores exist. For instance, concentrating on the Punjab map (Panel A), Northern districts are making a bunch of relatively healthier districts (Additional file 3, Figure B). Similarly, focussing on the KP map (Panel B), the districts from the upper part are making a cluster of deprived districts in terms of a composite index. Moreover, in Baluchistan province (Panel D), the districts near to the capital of Baluchistan (Quetta) are making a cluster of relatively healthy districts. The Sindh province (Panel C) has no specific clusters, however, the Central districts are relatively healthier than districts located at the boundary of the province.

The present study faced several limitations. First, there may be a large number of factors related to health but we have used only eight indicators from five different dimensions to construct a composite index (CHI), as the data set utilized in our study does not have rich information about other than the selected indicators. Secondly, the analysis was conducted only for 113 districts of Pakistan, and the rest of the districts were ignored either due to law and order situations or having fewer respondents (households) that could not show the real picture of the whole district. Finally, rich information was available on Islamabad district but in the present study, we have ignored this district. It is because Islamabad is the capital of Pakistan and after estimating the CHI score for that district, it was highly developed and was far away from all other study districts which made our results.

## Conclusion

To conclude here, spatial inequalities concerning access to health and health-related social indicators persist across countries and geographical regions within the country. In this regard, this study provides a foundation for measuring the extent of inequalities and heterogeneities within communities/districts. Besides, this analysis also offers a basis for assessing the spatial patterns of disparities across various geographical boundaries. Governments and public health researchers/practitioners can use such outcomes to set priorities and can eradicate the disparities as health-related socioeconomic attributes are integrated and improvement in any of these indicators especially in deprived/unhealthy regions will enhance the overall quality of health/wellbeing [23].

## Supplementary Information

**Additional file 1.** Steps of constructing choropleth map using ArcGIS application.

**Additional file 2:** **Table A.** List of upper and lower decile districts of Pakistan with respect to CHI scores. **Table B.** Regression analysis for disparity slope (Overall Pakistan). **Table C.** Region-wise list of upper and lower decile districts of Pakistan with respect to CHI scores. **Table D.** Regression analysis for disparity slope (Urban and Rural regions). **Table E.** Regression analysis for disparity slope (All provinces of Pakistan).

**Additional file 3:** **Figure A.** Distribution of Community Health Index of districts in all Provinces. **Figure B.** Spatial distribution of CHI of districts in all provinces of Pakistan.

## Data Availability

The Pakistan Social and Living Standard Measurement (PSLM) survey, Round-VI, 2014–15 dataset is publicly available to all users on the Pakistan Bureau of Statistics (PBS) website provided in the manuscript. The interested users can also access this dataset from the link: http://www.pbs.gov.pk/content/pakistan-social-and-living-standards-measurement

## Abbreviations

CHI: Community Health Index
HDI: Human Development Index
KP: Khyber Pakhtunkhwa
LHW: Lady Health Worker
WHO: World Health Organization
UHI: Urban Health Index
OLS: Ordinary Least Square
PSLM: Pakistan Social and Living Standard Measurement
PBS: Pakistan Bureau of Statistics
MDGs: Millennium Development Goals
SPSS: Statistical Package for Social Sciences
GIS: Geographic Information System
