# Supplementary material for: Inequalities in health and health-related indicators: a spatial geographic analysis of Pakistan
Source: BMC Public Health. 2020 Nov 26;20:1800. doi: 10.1186/s12889-020-09870-4 (PMC7690118; doi:10.1186/s12889-020-09870-4)
Supplement: Supplementary file 1 — Additional file 1. Steps of constructing choropleth map using ArcGIS application. [file 12889_2020_9870_MOESM1_ESM.docx]

**Additional file. 1**

**Steps of constructing choropleth map using ArcGIS application.**

1. In case of no ArcGIS application, Download and install Geographic Information System (GIS) software from “Environmental Systems Research Institute (ESRI)” website.
2. To construct a map, ensure that GIS shapefile and the variable for which map is going to be construct are available. Both of these files must have common geo-locational IDs, census tract IDs or ZIP codes.
3. Open the desired shapefile in ArcMap and import Excel data file (for which a map is going to be construct) in ArcMap.
4. After opening these files in ArcMap, join/merge these files by right clicking on the “boundary shapefile” in “layers”. A popup window will open. Click on “join and relate” and then on “Join”. A join data window will open. Select a unique geo-locational ID and variable for which map is going to develop. Click on “Keep all record” and then click on “Validate join”.
5. After merging/joining these files, right click again on “boundary shapefile” in “layers” and then click on “Properties”. A “layer properties” window will open. Click on “Symbology tab”, click “Quantities” and then select “Graduate colours”.
6. In the “field value”, select the variable for which map is going to construct and favourite “color ramp”. Click on “Classify” and “classification window” will open. In “classification method” select “Quantile” and determine the number of “classes” and then click on “OK”.
7. In final step, click on Insert tab and add title, legends, north arrow selector etc. to the constructed map. Once the map is ready, export the map with desired format by selecting “File tab” and then “Export Map”.
