# Supplementary material for: Inequalities in health and health-related indicators: a spatial geographic analysis of Pakistan
Source: BMC Public Health. 2020 Nov 26;20:1800. doi: 10.1186/s12889-020-09870-4 (PMC7690118; doi:10.1186/s12889-020-09870-4)
Supplement: Supplementary file 2 — Additional file 2: Table A. List of upper and lower decile districts of Pakistan with respect to CHI scores. Table B. Regression analysis for disparity slope (Overall Pakistan). Table C. Region-wise list of upper and lower decile districts of Pakistan with respect to CHI scores. Table D. Regression analysis for disparity slope (Urban and Rural regions). Table E. Regression analysis for disparity slope (All provinces of Pakistan). [file 12889_2020_9870_MOESM2_ESM.docx]

**Additional file 2**

**Table A.** **List of upper and lower decile districts of Pakistan with respect to CHI scores**

| **S. No** | **Lower decile** | **CHI** | **Upper decile** | **CHI** |
| --- | --- | --- | --- | --- |
| **1** | Umerkot | 0.01 | Sialkot | 0.47 |
| **2** | Sherani | 0.02 | Haripur | 0.49 |
| **3** | Jhal Magsi | 0.03 | Abbottabad | 0.51 |
| **4** | Torghar | 0.03 | Attock | 0.51 |
| **5** | Lorlai | 0.04 | Hyderabad | 0.52 |
| **6** | Barkhan | 0.04 | Jhelum | 0.53 |
| **7** | Harnai | 0.04 | Quetta | 0.60 |
| **8** | Chagai | 0.05 | Rawalpindi | 0.61 |
| **9** | Awaran | 0.05 | Peshawar | 0.66 |
| **10** | Mosakhel | 0.05 | Karachi | 0.76 |
| **11** | Kohistan | 0.05 | Lahore | 0.77 |

**Source:** Author’s own computations based on “Pakistan Social and Living Standard Measurement survey”, 2014-15

**CHI:** Community Health Index (standardized) scores

**Table B.** **Regression analysis for disparity slope (Overall Pakistan)**

Dependent variable: CHI scores

| **Variable name** | **Coefficient** | **St: error** | **t-ratio** | **Sig:** |
| --- | --- | --- | --- | --- |
| **Constant** | 6.007 | 0.349 | 17.197 | 0.000 |
| **Rank** | 0.384 | 0.007 | 58.706 | 0.000 |
|  | R^2^ = 0.97  R^2^ (Adjusted) = 0.97  F-statistic = 3446.39  Sig. (F) = 0.000 | | | |

**Source:** Author’s own computations based on “Pakistan Social and Living Standard Measurement survey”, 2014-15

**CHI:** Community Health Index (standardized) scores

**Table C.** **Region-wise list of upper and lower decile districts of Pakistan with respect to CHI scores**

|  | **Urban** | | | | **Rural** | | | |
| --- | --- | --- | --- | --- | --- | --- | --- | --- |
| **S. No** | **Lower decile** | **CHI** | **Upper decile** | **CHI** | **Lower decile** | **CHI** | **Upper decile** | **CHI** |
| **1** | Umerkot | 0.02 | Sahiwal | 0.66 | Umerkot | 0.01 | Abbottabad | 0.50 |
| **2** | Upper Dir | 0.05 | Mastng | 0.67 | Barkhan | 0.01 | Nowshera | 0.52 |
| **3** | Musakhel | 0.08 | Jhelum | 0.67 | Jhal magsi | 0.02 | Gujrat | 0.52 |
| **4** | Jhal magsi | 0.09 | Lahore | 0.69 | Tharparkar | 0.03 | Peshawar | 0.53 |
| **5** | Harnai | 0.09 | Rawalpindi | 0.70 | Sherani | 0.03 | Attock | 0.54 |
| **6** | Kohlu | 0.10 | Attock | 0.71 | Sujawal | 0.03 | Malakand | 0.54 |
| **7** | Lorlai | 0.11 | Abbottabad | 0.71 | Torghar | 0.04 | Haripur | 0.57 |
| **8** | Gawadar | 0.11 | Kalat | 0.72 | Nasirabad | 0.04 | Karachi | 0.60 |
| **9** | Ziarat | 0.12 | D.G. khan | 0.74 | Thatta | 0.04 | Jhelum | 0.60 |
| **10** | Chitral | 0.13 | Peshawar | 0.75 | Lorlai | 0.04 | Lahore | 061 |
| **11** | --- | --- | --- | --- | Lasbela | 0.05 | Rawalpindi | 0.63 |

**Source:** Author’s own computations based on “Pakistan Social and Living Standard Measurement survey”, 2014-15

**CHI:** Community Health Index (standardized) scores

**Table D.** **Regression analysis for disparity slope (Urban and Rural regions)**

Dependent variable: CHI scores

|  | **Urban regions** | | | | **Rural regions** | | | |
| --- | --- | --- | --- | --- | --- | --- | --- | --- |
| **Variable name** | Coefficient | St: error | t-ratio | Sig: | Coefficient | St: error | t-ratio | Sig: |
| **Constant** | 25.232 | 1.012 | 24.939 | 0.000 | 1.566 | 0.840 | 1.863 | 0.066 |
| **Rank** | 0.448 | 0.021 | 21.681 | 0.000 | 0.434 | 0.016 | 27.092 | 0.000 |
|  | R^2^ = 0.85  R^2^ (Adjusted) = 0.85  F-statistic = 470.068  Sig. (F) = 0.000 | | | | R^2^ = 0.89  R^2^ (Adjusted) = 0.89  F-statistic = 733.950  Sig. (F) = 0.000 | | | |

**Source:** Author’s own computations based on “Pakistan Social and Living Standard Measurement survey”, 2014-15

**CHI:** Community Health Index (standardized) scores

**Table E.** **Regression analysis for disparity slope (All provinces of Pakistan)**

Dependent variable: CHI scores

|  | **Punjab** | | | | **Khyber Pakhtunkhwa** | | | |
| --- | --- | --- | --- | --- | --- | --- | --- | --- |
| **Variable name** | Coefficient | St: error | t-ratio | Sig: | Coefficient | St: error | t-ratio | Sig: |
| **Constant** | 0.164 | 0.008 | 20.549 | 0.000 | 0.101 | 0.011 | 9.376 | 0.000 |
| **Rank** | 0.263 | 0.013 | 19.532 | 0.000 | 0.352 | 0.018 | 19.666 | 0.000 |
|  | R^2^ = 0.94  R^2^ (Adjusted) = 0.93  F-statistic = 381.494  Sig. (F) = 0.000 | | | | R^2^ = 0.96  R^2^ (Adjusted) = 0.95  F-statistic = 386.738  Sig. (F) = 0.000 | | | |
|  | **Sindh** | | | | **Baluchistan** | | | |
| **Variable name** | Coefficient | St: error | t-ratio | Sig: | Coefficient | St: error | t-ratio | Sig: |
| **Constant** | 0.055 | 0.016 | 3.543 | 0.000 | -0.002 | 0.007 | -0.363 | 0.721 |
| **Rank** | 0.303 | 0.026 | 11.600 | 0.000 | 0.278 | 0.011 | 22.973 | 0.000 |
|  | R^2^ = 0.88  R^2^ (Adjusted) = 0.87  F-statistic = 134.556  Sig. (F) = 0.000 | | | | R^2^ = 0.96  R^2^ (Adjusted) = 0.96  F-statistic = 527.757  Sig. (F) = 0.000 | | | |

**Source:** Author’s own computations based on “Pakistan Social and Living Standard Measurement survey”, 2014-15

**CHI:** Community Health Index (standardized) scores
