# Supplementary material for: Inequalities in health and health-related indicators: a spatial geographic analysis of Pakistan
Source: BMC Public Health. 2020 Nov 26;20:1800. doi: 10.1186/s12889-020-09870-4 (PMC7690118; doi:10.1186/s12889-020-09870-4)
Supplement: Supplementary file 3 — Additional file 3: Figure A. Distribution of Community Health Index of districts in all Provinces. Figure B. Spatial distribution of CHI of districts in all provinces of Pakistan. [file 12889_2020_9870_MOESM3_ESM.docx]

**Additional file 3**

**Figure A.** **Distribution of Community Health Index of districts in all Provinces**


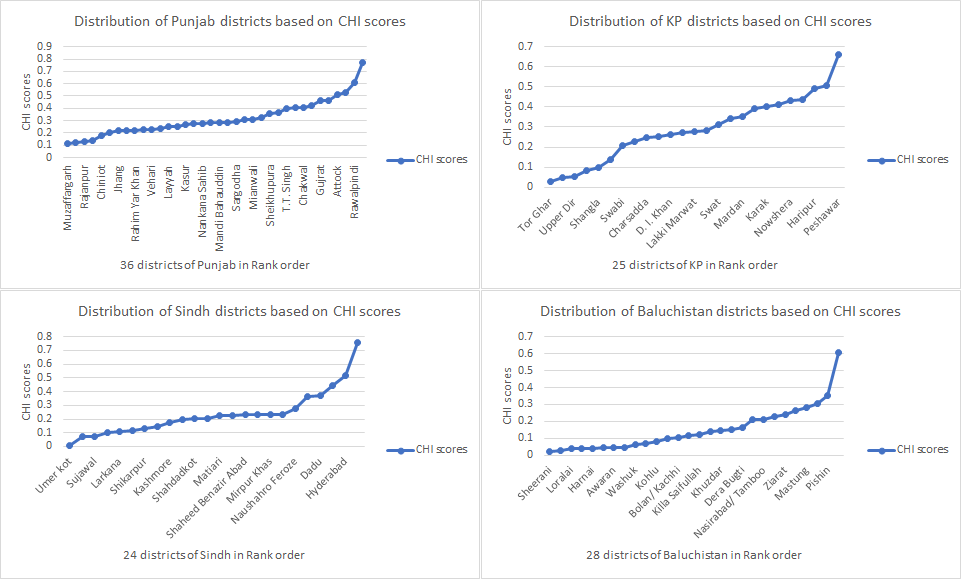


**Source:** Author’s own computations based on “Pakistan Social and Living Standard Measurement (PSLM) survey”, 2014-15

**CHI scores** are Community Health Index (Standardized) values

**Figure B.** **Spatial distribution of CHI of districts in all provinces of Pakistan**


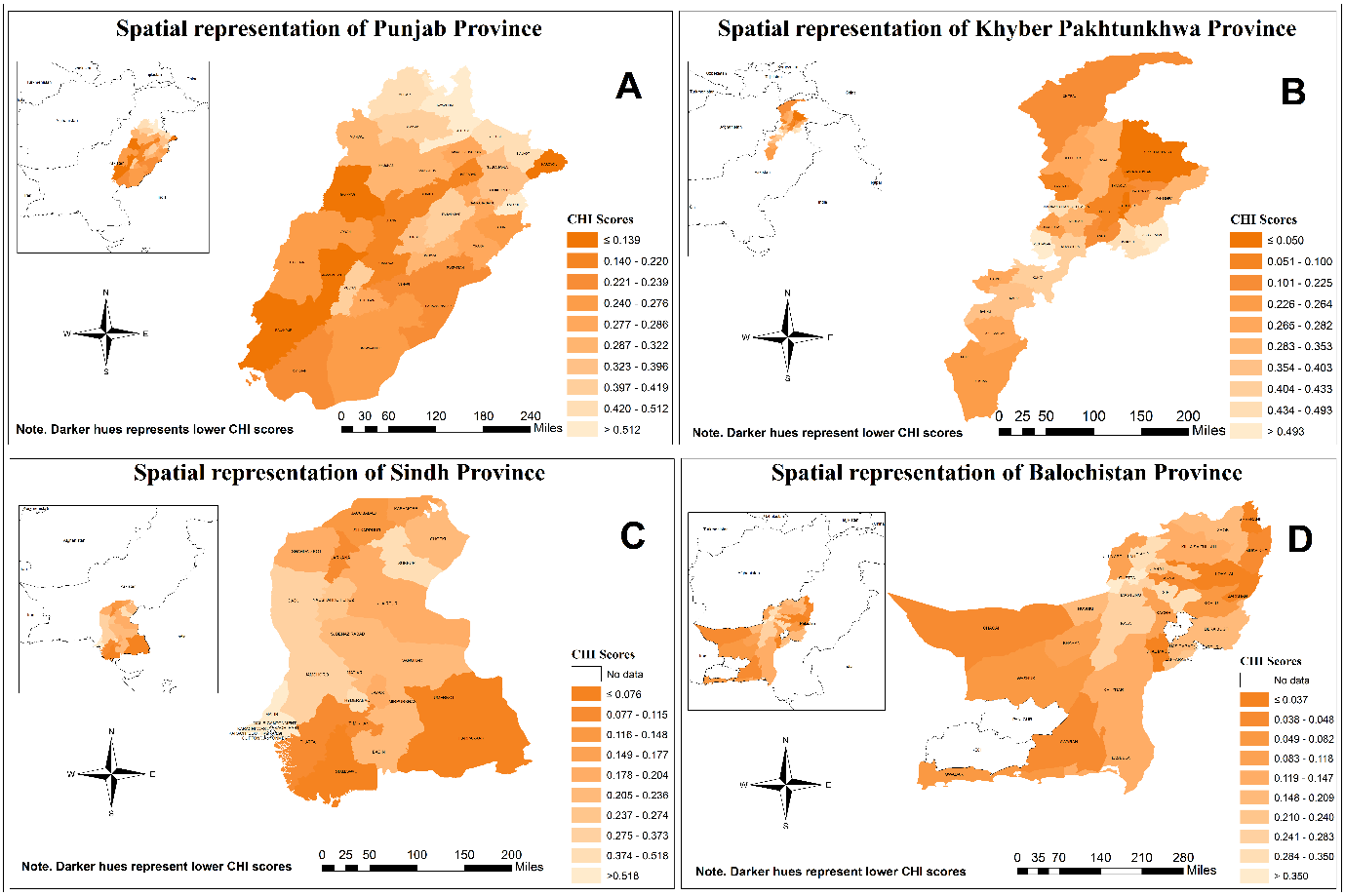


**Source:** Author’s own computations based on “Pakistan Social and Living Standard Measurement (PSLM) survey”, 2014-15. CHI scores are used to generated maps through ArcGIS, version 10.5 software.

Disparity ratios of 4.78, 12.51, 16.61, and 15.35 were estimated for Punjab, Khyber Pakhtunkhwa (KP), Sindh, and Baluchistan provinces respectively (Figure A). The ratio was relatively high in districts of Sindh province showing that upper and lower decile districts of Sindh were far away from one another. Moreover, “Lahore”, “Peshawar”, “Karachi” and “Quetta” were the top (healthiest/improved) districts concerning CHI scores respectively in Punjab, Khyber Pakhtunkhwa (KP), Sindh, and Baluchistan provinces. In contrast, “Muzaffarabad”, “Torghar”, “Umerkot” and “Sherani” were highly deprived districts of Punjab, Khyber Pakhtunkhwa (KP), Sindh, and Baluchistan provinces respectively. Besides, a higher disparity ingredient of 0.35 had been estimated for KP province, which depicts that comparatively, the districts of KP province were more heterogeneous (Additional file 2, Table E). By looking at the pattern in each province separately, it is obvious that districts of “Northern Punjab” were well-off/developed concerning CHI scores whereas districts of “Western Punjab” were worse-off/unhealthy (Figure B). “Lahore” and “Muzaffarabad” were the relatively developed and deprived districts of Punjab province. Considering other provinces, districts from “Northern KP”, “Eastern and Northern” districts of the Sindh, and “Northern and Western” districts of Baluchistan were relatively worse-off. Figure B has a detailed explanation of inequalities based on provinces.
